# Two sporadic cases of childhood-onset Hailey-Hailey disease with superimposed mosaicism

**DOI:** 10.1038/s41431-023-01316-w

**Published:** 2023-03-15

**Authors:** Yasuhiko Asahina, Umi Tahara, Satomi Aoki, Kazuhiko Nakabayashi, Chiharu Tateishi, Daisuke Hayashi, Masayuki Amagai, Daisuke Tsuruta, Akiharu Kubo

**Affiliations:** 1 Department of Dermatology, Keio University School of Medicine, Tokyo, Japan; 2 Department of Maternal-Fetal Biology, National Center for Child Health and Development, Tokyo, Japan; 3 Department of Dermatology, Osaka Metropolitan University, Medical School, Osaka, Japan; 4 Division of Dermatology, Department of Internal Related, Kobe University Graduate School of Medicine, Kobe, Japan

**Keywords:** Disease genetics, Genetic testing

## Abstract

A prenatal second-hit genetic change that occurs on the wild-type allele in an embryo with a congenital pathogenic variant allele results in mosaicism of monoallelic and biallelic defect of the gene, which is called superimposed mosaicism. Superimposed mosaicism of Hailey-Hailey disease (HHD) has been demonstrated in one familial case. Here, we report two unrelated HHD cases with superimposed mosaicism: a congenital monoallelic pathogenic variant of *ATP2C1*, followed by a postzygotic copy-neutral loss of heterozygosity. Uniquely, neither patient had a family history of HHD at the time of presentation. In the first case, the congenital pathogenic variant had occurred de novo. In the second case, the father had the pathogenic variant but had not yet developed skin symptoms. Our cases showed that superimposed mosaicism in HHD can lack a family history and that genetic analysis is crucial to classify the type of mosaicism and evaluate the risk of familial occurrence.

## Introduction

Hailey-Hailey disease (HHD; OMIM #169600) is a blistering dermatosis characterized by recurrent, erythematous, and vesicular plaques [1]. HHD is caused by a heterozygous germline variant in *ATP2C1* located on chromosome 3q22 [2]. Skin lesions usually begin to develop at 20–40 years of age. Lesions affect the whole body with aggravation in flexural areas [1].

In addition to typical cases of HHD, cases with skin lesions distributed along Blaschko’s lines have been reported. This indicates cutaneous segmental mosaicism (Table 1) [3–12]. Cutaneous segmental mosaicism of autosomal dominant disease can be classified into two types, according to the state of zygosity [13, 14]. Simple segmental mosaicism (type 1 segmental mosaicism) is caused by a prenatal postzygotic variant in the wild-type embryo [13]. Simple segmental mosaicism of HHD presents with Blaschko-linear skin lesions where the pathogenic keratinocytes are distributed. In contrast, superimposed mosaicism (type 2 segmental mosaicism) is caused by a prenatal second-hit genetic change that occurs on the wild-type allele in an embryo with a congenital pathogenic variant allele [13]. Superimposed mosaicism of HHD presents as aggravated skin lesions in a Blaschko-linear pattern where biallelic mutant keratinocytes are distributed.

**Table 1:** Reported cases of Hailey-Hailey disease with mosaic manifestation.

Case (reference)	Age	Sex	Onset	Family history	Distribution of skin lesions	Congenital and postzygotic genetic changes in *ATP2C1*	Type of mosaicism
1 [3–5]	5 years	F	3 months	+	Unilateral, later bilateral in body folds	Congenital monoallelic c.2126+1G>A variant and postzygotic cn-LOH	Superimposed (type 2) mosaicism
2 [6]	81 years	M	Childhood	−	Unilateral	NA	
3 [7]	70 years	F	Childhood	−	Unilateral	NA	
4 [8]	4 years	M	6 months	−	Unilateral	NA	
5 [9]	60 years	M	Earlier than 20 years	+	Bilateral in body folds, and unilateral in the trunk and extremities	NA	
6 [10]	8 months	F	1 month	−	Linear unilateral	NA	
7 [11]	47 years	F	40 years	−	Linear unilateral	Postzygotic monoallelic c.238A>T (p.Lys80*) variant	Simple segmental (type 1) mosaicism
8 [12]	72 years	M	Elderly	−	Linear unilateral	Postzygotic monoallelic c.1402C>T (p.Arg468*) variant	Simple segmental (type 1) mosaicism

Nucleotide change in *ATP2C1* and the predicted amino acid change are described based on NM_001378687.1 and NP_001365616.1, respectively.

*NA* not available, *cn-LOH* copy neutral-loss of heterozygosity, *a premature termination codon.

Both childhood-onset and adult-onset cases of mosaic HHD have been reported. In two adult-onset cases in patients with no family history of HHD, a de novo pathogenic variant was detected in skin lesions, indicating simple segmental mosaicism [11, 12]. In contrast, one case of childhood-onset mosaic HHD occurred in a patient with a family history of HHD; the patient exhibited a congenital pathogenic *ATP2C1* variant with a loss of heterozygosity (LOH) regarding variation in the linear skin lesion, indicating superimposed mosaicism [3–5]. Here, two cases of childhood-onset HHD are presented. Both cases involved a linear distribution of skin lesions in patients with no family history of the disease at the time of presentation.

## Materials and methods

### Sample preparation

Sample collection was approved by the Ethics Committee of Keio University School of Medicine and performed after obtaining written informed consent. In case 1, the skin specimen was obtained by biopsy and the epidermis was separated from the dermis by dispase treatment, as described previously [15]. In case 2, the blister roof was collected. Genomic DNA was obtained from the epidermis and peripheral blood leukocytes using a Maxwell RSC Instrument and Maxwell RSC Blood DNA Kit (Promega, Madison, WI).

### Targeted exome sequencing

Targeted exome sequencing, which was designed to analyze ~500 causative genes of genodermatoses, was performed as previously described [15, 16]. In brief, DNA libraries were prepared using HaloPlex target enrichment system (Agilent technologies, Santa Clara, CA) and were sequenced using HiSeq 2500 system (Illumina, San Diego, CA).

### Sanger sequencing of the genomic DNA

Genomic DNA amplification and subsequent direct Sanger sequencing of *ATP2C1* were performed using the primer pairs shown in Supplementary Table S1.

### Single nucleotide polymorphism BeadChip analysis

DNA amplification, labeling, and hybridization were performed according to the manufacturer’s instructions using the HumanCytoSNP-12 v2.1 DNA Analysis BeadChip Kit (WG-3202103; Illumina). The array slides were scanned on an iScan system (Illumina), and log R ratios and B-allele frequencies were calculated and visualized using KaryoStudio data-analysis software (ver. 1.4; Illumina).

## Results

### Clinical case presentation

**Fig. 1:**
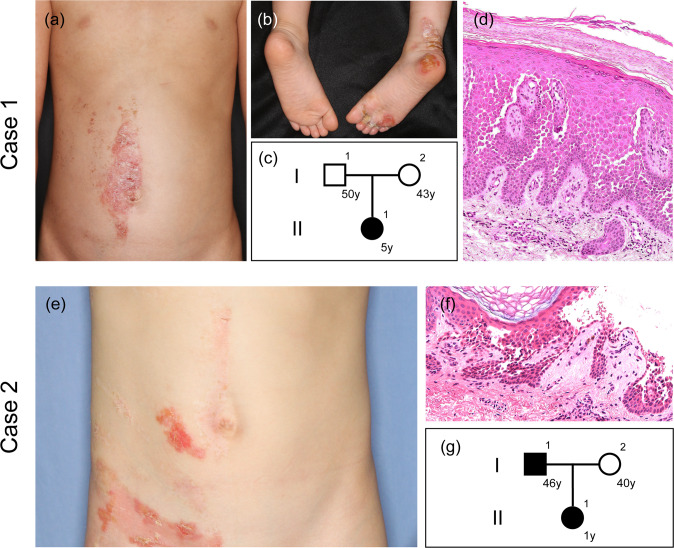
Clinical and histopathological features. Case 1 (**a**–**d**) and case 2 (**e**–**g**). **a**, **b** Red plaques with scales on the right side of the abdomen and foot. Blisters are also evident in the sole. **e** Erosions and blisters on the right side of the abdomen and groin. **c**, **g** Family tree. **d**, **f** Suprabasal acantholysis with a dilapidated brick wall appearance (original magnification ×400). y years.

The first case (case 1) involved a 5-year-old Japanese girl with recurrent red plaques and scales on the abdomen, buttock, labia, popliteal fossa, and foot (Fig. 1a, b). Her age at onset was 3 years old. The skin lesions were distributed only on the right side of the body. The patient had an unremarkable family history (Fig. 1c). The father and mother were 50 and 43 years old, respectively. A skin biopsy from the patient revealed a dilapidated brick wall appearance of basal cells with suprabasal clefts and acantholytic cells (Fig. 1d).

The second case (case 2) involved a 10-year-old Japanese girl who had recurrent erosions and blisters on her arm, axilla, and abdomen since birth. The Blaschko-linear skin lesions were distributed only on the right side (Fig. 1e). A skin biopsy from the patient revealed a suprabasal cleft with acantholytic cells (Fig. 1f). Neither parent had any skin symptoms. However, 6 years after the first visit, the father developed erosions in the bilateral axilla and scrotum at the age of 46 years (Fig. 1g).

A clinical diagnosis of mosaic HHD was made in both cases. To establish the diagnosis and elucidate the underlying mechanism of mosaic manifestation, we performed a genetic analysis after obtaining written informed consent approved by the Institutional Review Board of Keio University.

### Genetic analysis results

Targeted exome sequencing was performed on genomic DNA purified from epidermis. We identified *ATP2C1* (NM_001378687.1) heterozygous variants including the c.1940A>T (p.Asn647Ile) variant in case 1 and the c.2104T>A (p.Phe702Ile) variant in case 2. These *ATP2C1* variations have not been registered in single nucleotide polymorphism databases (ClinVar, gnomAD and UniProt) [17–19]. S-VAR (http://p4d-info.nig.ac.jp/s-var/; a prediction tool that uses PolyPhen-2, SIFT, PROVEAN, and PANTHER) indicated that both variants were pathogenic or caused deleterious changes (Supplementary Table S2). The case 1 and case 2 variants are classified as “likely pathogenic” and “uncertain significance”, respectively, according to the American College of Medical Genetics and Genomics guideline for classifying sequence variants [20].

**Fig. 2:**
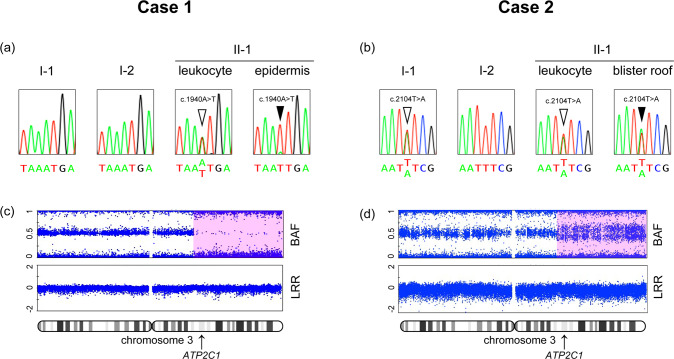
Genetic analyses of *ATP2C1*. Case 1 (**a**, **c**) and case 2 (**b**, **d**). **a** Sanger sequencing chromatograms showing the heterozygous c.1940A>T variant (open arrowhead) and the homozygous c.1940A>T variant (filled arrowhead). **b** Sanger sequencing chromatograms showing the heterozygous c.2104T>A variant (open arrowhead). The nucleotide peak of A is higher than that of T in the same nucleotide site (filled arrowhead). **c**, **d** Single nucleotide polymorphism array analyses of chromosome 3. B-allele frequency of chromosome 3 in the epidermis showing loss of heterozygosity (pink square). BAF B allele frequency, LRR log R ratio.

Sanger sequencing confirmed the heterozygous c.1940A>T variant in case 1 (Fig. 2a) and the c.2104T>A variant in case 2 (Fig. 2b). The father of the patient in case 2 had the c.2104T>A variant, whereas the variant in case 1 was a de novo mutation. Sanger sequencing of genomic DNA purified from the lesional epidermis of case 1 suggested a LOH of the variant allele (Fig. 2a). In case 2, the nucleotide peak of the variant allele was higher than that of the wild-type allele in the blister roof, compared with peripheral leukocytes, suggesting lesion-specific LOH (Fig. 2b).

LOH is induced by several causes including chromosome recombination, chromosomal deletion, and de novo occurrence of an identical variant on the wild-type allele. Next, a single nucleotide polymorphism array analysis was performed. In both cases, the B allele frequency (BAF) revealed a LOH beginning proximal to *ATP2C1* and extending to the telomere on chromosome 3q, while the logR ratio indicated a normal DNA copy number over the entire length of chromosome 3 (Fig. 2c, d). The BAF deviation was small in case 2, in which the blister roof was sampled (Fig. 2d). It is probably because the keratinocytes in the blister roof had LOH but in low numbers, and many extravascular immune cells without LOH that were present in the blister fluid were mixed in the sample.

These results indicated that the epidermal cells of the lesional skin had acquired copy-neutral LOH (cn-LOH) on chromosome 3q that converted the variant of *ATP2C1* from heterozygous to homozygous in both patients.

## Discussion

Superimposed mosaicism results in segmental lesions with more severe skin manifestations [13, 14]. The two cases presented in this study and a previous case (case 1 in Table 1) revealed that superimposed mosaicism of HHD showed linearly distributed severe skin lesions with onset during childhood because of *ATP2C1* biallelic defects [3–5]. The reported mosaic HHD cases implied that the clinical course of HHD differs depending on the type of mosaicism. In brief, simple segmental mosaicism of HHD results in the development of Blaschko-linear skin lesions in adults. Superimposed mosaicism of HHD results in Blaschko-linear skin lesions that begin early in life and continue for a lifetime, with the general skin symptoms of HHD developing later in life. Further accumulation of mosaic HHD cases is needed to evaluate the relationship between age at onset and the type of mosaicism.

To date, simple segmental mosaicism without a family history and superimposed mosaicism with a family history have been genetically confirmed in cases of HHD. The two cases of superimposed mosaicism investigated in this study had no family history of HHD at the time of presentation. The superimposed mosaicism in case 1 was caused by a germline de novo variant, followed by cn-LOH. Thus, case 1 was a true sporadic case. Case 2 had no obvious family history at the first visit, but the father later developed typical HHD skin lesions. Several reported cases of childhood-onset HHD with mosaic manifestation were diagnosed as, or suspected to be, simple segmental mosaicism based on the lack of family history, without genetic testing [7, 10]. Conversely, the clinical courses of those cases imply a diagnosis of superimposed mosaicism. Diagnosis of simple segmental and superimposed mosaicism based on family history alone should be avoided.

It is important to determine the type of mosaicism to evaluate the risk of familial disease occurrence of HHD. In simple segmental mosaicism, there is no risk of occurrence in a patient’s sibling. The risk of occurrence of HHD in offspring depends on whether the patient’s germ cells are in a mosaic state. In contrast, in superimposed mosaicism, the risk of occurrence of HHD in a patient’s offspring is approximately 50%. Genetic testing in superimposed mosaicism can determine whether the germline pathogenic variant was inherited from a parent or occurred de novo, which determines the risk of occurrence in a patient’s sibling. The cases in this study highlight the importance of detailed genetic analysis to classify the type of mosaicism and provide information regarding familial risk.

## Supplementary information

Table S1, S2

## Data Availability

The data that support the findings of this study are available on request from the corresponding author. The data are not publicly available due to privacy or ethical restrictions.
