# Supplementary material for: Two sporadic cases of childhood-onset Hailey-Hailey disease with superimposed mosaicism
Source: Eur J Hum Genet. 2023 Mar 15;31(6):716–20. doi: 10.1038/s41431-023-01316-w (PMC10250405; doi:10.1038/s41431-023-01316-w)
Supplement: Supplementary file 1 — Table S1, S2 [file 41431_2023_1316_MOESM1_ESM.docx]

Table S1. Primer list.

| Patient | Forward primer | Reverse Primer |
| --- | --- | --- |
| Case 1 | 5’-TCATTGAACAATGGGTGGTC-3’ | 5’-TTCCAGCCACACAAATCATC-3’ |
| Case 2 | 5’-CCAGGTGATCTGCCAGTTTC-3’ | 5’-GAGGAAACAATGAGATGTGAGC-3’ |

Table S2. Prediction of pathogenicity of the *ATP2C1* variants.

| Patient | Nucleotide change (predicted amino acid change) | PolyPhen-2 | SIFT | PROVEAN | PANTHER |
| --- | --- | --- | --- | --- | --- |
| Case 1 | c.1940A>T  (p.Asn647Ile) | 1.0  (probably damaging) | 0.0  (deleterious) | -8.662  (deleterious) | -6.96775  (deleterious) |
| Case 2 | c.2104T>A  (p.Phe702Ile) | 1.0  (probably damaging) | 0.02  (deleterious) | -5.62  (deleterious) | -3.83812  (deleterious) |
